# Different feed efficiency modeling approaches for the prediction of genomic breeding values in lactating dairy cows

**DOI:** 10.3389/fgene.2026.1815864

**Published:** 2026-04-15

**Authors:** A. Chegini, E. Negussie, T. Kokkonen, M. H. Lidauer

**Affiliations:** 1 Natural Resources Institute Finland (Luke), Jokioinen, Finland; 2 Department of Agricultural Sciences, University of Helsinki, Helsinki, Finland

**Keywords:** feed efficiency, prediction accuracy, regression on expected feed intake, residual feed intake, single-step genomic best linear unbiased prediction

## Abstract

Feed is the main cost of production in dairy farming. Any improvement in feed efficiency (FE) would increase marginal profit and sustainability and mitigate the environmental impact of dairy farming. In this study, we applied single-step genomic best linear unbiased prediction to different feed-efficiency metrics using records collected from Nordic Red dairy cattle (RDC). The main objective was to compare different metrics in terms of their effectiveness in selecting more feed-efficient animals. Weekly observations (n = 22,071) of dry-matter intake records from 791 RDC cows collected from 1998 to 2021 were used in this study. The pedigree consisted of 5,604 individuals, of which 1,489 animals were genotyped. Different modeling approaches, including conventional residual feed intake (RFI), regression on expected feed intake (ReFI), two multi-trait residual feed efficiency indices (RFI_Index_ and RZFE), and energy conversion efficiency (ECE) were analyzed. For the ReFI approach, two alternatives for predicting the expected feed intake, namely, a prediction equation tailored to the RDC data and a prediction equation based on Holstein dairy cow data proposed by the National Academies of Sciences, Engineering, and Medicine (NRC 2021), were compared. First, a BLUP model was developed, and the necessary variance components were estimated for each approach. Then, pedigree-based and genomic-enhanced breeding values (PEBV and GEBV, respectively) were estimated using either reduced or full datasets. For model validation, PEBV and GEBV estimated using the full dataset were regressed on PEBV and GEBV estimated using the reduced dataset, respectively, to measure bias, dispersion, and prediction accuracy (PAC). The heritability estimates of different residual metrics ranged from 0.23 for RFI to 0.30 for ReFI_NRC2021_, and the repeatability estimates ranged from 0.48 to 0.52. The estimated heritability and repeatability of ECE were 0.23 and 0.56, respectively. For all metrics, the use of genomic information increased PAC. However, there were discrepancies between the metrics in terms of the magnitude of PAC, with the PAC being the highest for ReFI_RDC_ and the lowest for RFI_Index_. Similarly, ReFI_RDC_ had the lowest bias, while the highest bias was estimated for RFI_Index_. In addition, RZFE and ReFI_RDC_ showed lower dispersion. The correlations between GEBV of the residual metrics and the GEBV of ECE were lowest for RFI_NRC2021_ and RFI and highest for ReFI_RDC_. Among the metrics compared, ReFI_RDC_ and RFI_Index_ showed the highest effectiveness in selecting efficient cows. This indicates that the use of appropriate partial regression coefficients and the type of modeling are vital in breeding programs aimed at enhancing FE.

## Introduction

Feed cost is the main cost of production in dairy farming; in some production systems, it accounts for up to 75 percent of total production costs (Alqaisi et al., 2011). Therefore, any improvement in the efficiency of feed utilization would improve the profitability of dairy farmers and reduce the emission of greenhouse gases (Negussie et al., 2017). Indirect improvements in feed efficiency (FE) have been observed over the last decades as a consequence of genetic selection for milk yield. This is because by increasing milk yield, maintenance requirement is diluted, and consequently, a higher proportion of energy consumption is allocated to production (Capper et al., 2008). However, it has been stated that further improvement in FE through increased milk yield is difficult as there is uncertainty whether the dilution of maintenance evolves linearly or curvilinearly (VandeHaar and St-Pierre, 2006; Bach et al., 2020). In addition, further selection for increased milk yield can impair fertility and the health status of the animal (Pryce et al., 2004; Oltenacu and Broom, 2010).

Different studies analyzed the possibility of improving FE in different species (i.e., pig, chicken, turkey, and beef), especially in dairy cattle in the last decade (Pryce et al., 2012; Berry and Crowley, 2013; Abdalla et al., 2019; Islam et al., 2020; Wang et al., 2022; Lidauer et al., 2023). Different metrics have been studied to improve FE, including feed conversion ratio, gross energy efficiency, and energy conversion efficiency (ECE) (Liinamo et al., 2015). Although they are easier to calculate and interpret, the ratio traits were not found to be proper metrics for FE because selection for their improvement may impose inequivalent selection pressure on the component traits (Gunsett, 1984). Moreover, they are highly correlated with their components and can increase error variance in the statistical analysis (Berry and Crowley, 2013). Among the suggested metrics, residual feed intake (RFI) is the most studied and well-known metric to improve FE, and it is calculated by subtracting the expected dry matter intake (eDMI) from the actual dry matter intake (DMI) (Koch et al., 1963). Conventionally, eDMI is calculated by regressing DMI on energy sinks, mainly energy corrected milk (ECM), metabolic body weight (MBW), body weight (BW) loss, and BW gain.

Kennedy et al. (1993) stated that RFI is correlated with energy sinks and has some disadvantages; therefore, they proposed a metric that is uncorrelated with energy sinks, known as the genetic RFI (gRFI). However, it was found that gRFI has similar features to RFI in finding feed-efficient animals. This is because the estimated partial phenotypic regression coefficients that model the eDMI in gRFI resemble the estimated partial regression coefficients of RFI (Lu et al., 2015; Lidauer et al., 2023). Lu et al. (2015) stated that the calculation of RFI is based on the assumption that the recording of energy sinks is without measurement errors and that regression coefficient estimates are not confounded. However, both assumptions may not be met and could yield illogical partial regression coefficients for the calculation of eDMI. For instance, the partial regression coefficient for ECM was estimated to be below 0.30 in several studies (Tempelman et al., 2015; Li et al., 2017; Lidauer et al., 2023). Considering the metabolizable energy (ME) required for producing 1 kg of ECM (4,765 kJ/kg; National Academies of Sciences, Engineering, and Medicine, 2021), this means that the energy content of the feed should be at least 15,883 kJ/kg DMI because otherwise the cows’ efficiency of using energy for lactation would be greater than 100 percent. To address this discrepancy, Lidauer et al. (2023) suggested calculating eDMI and deriving partial regression coefficients from energy requirement formulations that were estimated in nutritional studies. They have also demonstrated that regressing DMI on eDMI, i.e., applying the so-called “regression on expected feed intake (ReFI)” metric, would lead to the selection of more feed-efficient animals.

The objectives of this study were to 1) estimate variance components and the pedigree-based and genomic-enhanced breeding values (PEBV and GEBV) for five different feed utilization efficiency metrics and 2) compare the different metrics in terms of their effectiveness and accuracy in selecting feed-efficient animals by applying forward validation and calculating correlations of GEBV among the five different metrics with GEBV and adjusted phenotypes of ECE, which serves as a measurement of the realized overall FE of a lactating cow.

## Materials and methods

### Data

The data used in this study were extracted from the FE research database of the Natural Resources Institute Finland (Luke). All the animals enrolled in the feed-intake recording were primiparous Nordic Red dairy cattle (RDC), the majority of which were recruited from the VikingGenetics nucleus breeding herd and are, therefore, genetically superior cows of the Nordic RDC population. Recording was carried out at four different research farms: two in Jokioinen and one each in Helsinki and Maaninka. The cows at the latter two farms were housed in free-stall barns, while those at the Jokioinen herd were housed in a tie-stall barn until 2009 and in a free-stall barn thereafter.

All cows included in the data were fed *ad libitum*, and the diets were based on grass silage and a home-blend concentrate mixture, which contained an average of 48% concentrate in DM. For the chemical analyses of the diets, samples of grass silages were taken twice a week and combined into 4-week samples, and concentrate samples were taken once a week and combined into 6-week samples. Milk samples were analyzed once a week until lactation week 8 and monthly until the end of lactation using infrared analyzers (MilkoScan FT 6000, Foss, Hillerød, Denmark) at the Valio Ltd. milk laboratory (Seinäjoki, Finland). Data collection started 5 days after calving and continued throughout the whole lactation period, up to a maximum of 305 days in milk (DIM). Feed intake was not recorded during the pasture period; therefore, cows that calved at the end of the calving period (September to December) had gaps in their feed-intake records. The BW observations used in this study were calculated by fitting cow-specific random regression functions to the BW measurements (Mäntysaari and Mäntysaari, 2015) and predicting daily BW and daily BW change observations from the fitted functions.

**FIGURE 1:**
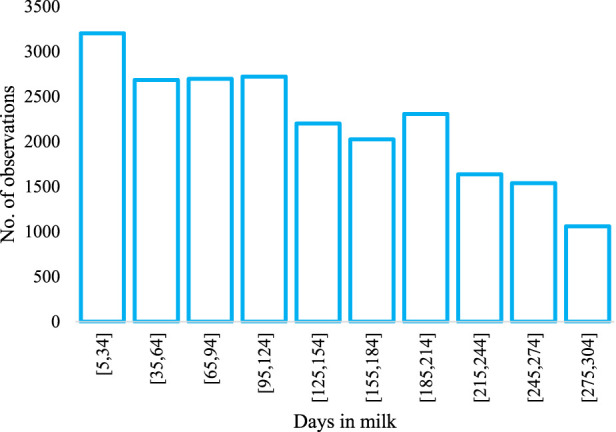
Distribution of dry matter intake records throughout lactation in the dataset.

The raw data consisted of 138,365 weekly and daily observations, from which 22,071 weekly averages for 791 individuals were formed and used for this study. Phenotypic records were available from 1998 to 2021. Descriptive statistics for the different traits are presented in Table 1. The formula stated by Sjaunja et al. (1990) was used to calculate ECM, i.e., ECM = milk yield × [(38.30 × fat content +24.20 × protein content +16.54 × lactose content +20.7)/3,140]. The ECE observations were calculated by dividing the ECM observations by the metabolizable energy intake observations. The distribution of phenotypic records within 30-day intervals throughout lactation is shown in Figure 1.

**TABLE 1:** Descriptive statistics of the phenotypic records.

Trait^a^	n	Mean	S.D.	Min	Max	CV (%)
DMI (kg)	22,071	19.4	3.05	8.40	30.96	15.70
MEI (MJ/d)	22,060	212.7	30.49	103.53	328.20	14.33
ECM (kg)	22,032	29.0	4.46	11.58	45.85	15.40
MBW	22,063	119.7	8.91	85.40	156.72	7.45
BWL	4,261	0.35	0.37	0.00	1.70	105.71
BWG	17,267	0.35	0.23	0.00	1.29	65.71
BCS	22,050	3.17	0.33	1.75	4.85	10.36
eDMI_RDC_	21,513	20.0	2.28	7.12	29.98	11.38
eDMI_NRC2021_	22,003	20.8	1.94	12.49	29.35	9.31
eDMI_FIT_	21,524	19.6	1.97	8.35	27.50	10.06
ECE (kg/MJ)	21,954	0.137	0.022	0.060	0.218	15.66

^a^ DMI, dry matter intake; MEI, metabolizable energy intake; ECM, energy-corrected milk; MBW, metabolic body weight (BW_0.75_); BWL, body weight loss; BWG, body weight gain; BCS, body condition score; eDMI_RDC_, expected DMI calculated using partial regression coefficients tailored to the RDC data; eDMI_NRC2021_, expected DMI calculated using the DMI predicting equation from National Academies of Sciences, Engineering, and Medicine (2021); eDMI_FIT_, expected DMI calculated by fitting a single-step residual feed intake model; ECE, ECM/metabolizable energy intake (energy conversion efficiency).

Cows with observations were the daughters of 309 sires and 666 dams, of which 289 sires and 229 dams were genotyped, and from 2004 onwards, 431 cows with FE observations were genotyped. In total, 1,489 animals (762 male and 727 female) were genotyped using the Illumina BovineSNP50 BeadChip (Illumina, San Diego, CA). Using the same edition criteria as the Nordic Cattle Genetic Evaluation (NAV), 46,914 SNP markers remained. The pedigree was pruned for cows with observations by including up to five generations, resulting in a total of 5,604 animals. Animals with unknown parents were assigned to 10 unknown parent groups according to their birth year and origin to account for genetic differences because all cows in the Jokioinen herd were recruited from the RDC nucleus breeding program and were closely related to the RDC elite bull population.

### Statistical analysis

Five different feed-utilization efficiency metrics were studied.

#### Regression on the expected feed intake

The first two metrics were based on the ReFI approach (Equation 1) and were modeled using a random regression model as follows:

$${\text{DMI}}_{\text{himwp}}=\alpha_{h}\times {\text{eDMI}}_{\text{iw}}+\beta_{m}\times {\text{eDMI}}_{\text{iw}}+\gamma_{\text{PEi}}\times {\text{eDMI}}_{\text{iw}}+\delta_{\text{Ai}}\times {\text{eDMI}}_{\text{iw}}+e_{\text{himwp}},$$ (1)

where DMI_himwp_ is a DMI observation of cow i in lactation week w and lactation period class p recorded in the herd × production-year (HPY) h and herd × trial × test month (HTM) m with 791, 43, 5, 28 and 261 effect levels, respectively; α_h_ is a fixed regression coefficient nested within HPY; β_m_ is a random regression coefficient nested within HTM [**β** ∼ N(**0**, **I** × $\sigma_{\beta }^{2}$ ), where **I** is an identity matrix and $\sigma_{\beta }^{2}$ is the variance of the HTM coefficients]; γ_PEi_ is the random regression coefficient for the animal permanent environmental effect [**γ** ∼ N(**0**, **I** × $\sigma_{\gamma }^{2}$ ), where **I** is an identity matrix and $\sigma_{\gamma }^{2}$ is the variance of the permanent environmental regression coefficients]; δ_Ai_ is the random regression coefficient for the animal additive genetic effect with [**δ** ∼ N(**0**, **A** × $\sigma_{\delta }^{2}$ )] or [**δ** ∼ N(**0**, **H** × $\sigma_{\delta }^{2}$ )], where $\sigma_{\delta }^{2}$ is the variance of the additive genetic regression coefficient, **A** is the pedigree-based relationship matrix, and **H** is the combined pedigree and genomic relationship matrix that will be explained later, respectively; and e_himwp_ is the residual random effect [**e** ∼ N(**0**, **R**), where $R=\sum_{p=1}^{5}⊕I_{\left(p\right)}\sigma_{e_{\left(p\right)}}^{2}$ ] and considering five lactation period classes (lactation month 1, 2, 3, 4–8, and 9–11).

For calculating the expected dry-matter intake (eDMI_iw_) for cow i that was in lactation week w, we applied two energy requirement formulations. The first formulation was developed based on the RDC FE data (Lidauer et al., 2023) and considers four energy sinks:

$${\text{eDMI}}_{\text{iw}}\;\left(\text{kg}/d\right)=0.456\times {\text{ECM}}_{\text{iw}}+0.0508\times {\text{MBW}}_{\text{iw}}\;–\;3.25\times {\text{BWL}}_{\text{iw}}+3.25\times {\text{BWG}}_{\text{iw}},$$

where BWL and BWG are the BW loss and BW gain, respectively. The second formulation was recommended by the National Academies of Sciences, Engineering, and Medicine (2021) for predicting DMI in Holstein cows and was tailored for first parity cows:

$${\text{eDMI}}_{\text{iw}}\;\left(\text{kg}/d\right)=\left[3.7+0.305\times {\text{MilkE}}_{\text{iw}}+0.022\times {\text{BW}}_{\text{iw}}\;–\;0.689\times {\text{BCS}}_{\text{iw}}\right]\times \left[1\;–\;\left(0.212\right)\times e^{\left(-0.053\times \;\mathrm{DIM}\right)}\right],$$

where MilkE is milk energy (Mcal/d) and BCS is the body condition score. From here onwards, **ReFI**_**RDC**_ refers to the ReFI metric based on the first formulation, and **ReFI**_**NRC2021**_ refers to the ReFI metric based on the second formulation.

#### Residual feed intake index

As an alternative to the conventional RFI, a breeding value (BV) for RFI based on an index was developed (**RFI**_**Index**_). First, the breeding values were predicted from a multi-trait (i.e., Equation 2) model that included five traits, namely, DMI, ECM, MBW, BWL, and BWG, as follows:

$${\text{DMI}}_{t:\text{ihmlwp}}=\sum_{k=1}^{5}\gamma_{t:\text{lk}}\;{Ø}_{\text{iwk}}+{\text{HPY}}_{t:h}+{\text{HTM}}_{t:m}+{\text{pe}}_{t:i}+u_{t:i}+e_{t:\text{ihmlw}p},$$ (2)

where DMI_t:ihmlwp_ is the observation for trait t of cow i made in the lactation week w in HPY h within the contemporary group m; $\sum_{k=1}^{5}\gamma_{t:\text{lk}}\;{Ø}_{\text{iwk}}$ is the fixed regression function on lactation week w nested within herd l, where ${Ø}_{iwk}$ is the *k*th coefficient of a fourth-order Legendre polynomial; HTM_t:m_ is the random effect of HTM m on trait t with [var(HTM) ∼ MVN (**0**, **I** ⊗ **M**), where **I** is an identity matrix and **M** is a 5 × 5 matrix of HTM (co)variances] and ⊗ is the Kronecker product; pe_t:i_ and u_t:i_ are the animal permanent environmental and animal additive genetic effects, respectively, for trait t with [var(**pe**) ∼ MVN (**0**, **I** ⊗ **P**), where **I** is an identity matrix, **P** is a 5 × 5 matrix of permanent environmental (co)variances], and with [var(**u**) ∼ MVN (**0**, **A** ⊗ **V**_**a**_) or var(**u**) ∼ MVN (**0**, **H** ⊗ **V**_a_), where **A** is the pedigree-based relationship matrix or **H** is the combined single-step relationship matrix, respectively, and **V**_a_ is a 5 × 5 matrix of animal additive genetic (co)variances]; and e_t:ihmlwp_ is the random residual effect with [var(**e**) ∼ MVN (**0**, **R**), where **R** is the (co)variance matrix for the residual effects, $R=\sum_{p=1}^{5}⊕I_{\left(p\right)}R_{\left(p\right)}$ , considering five lactation period classes].

In the second step, for each animal i, a pedigree-based or single-step based BV index, (i.e., RFI_Index_ ; Equation 3), was derived by applying the same index weights as used for ReFI_RDC_ to an animal’s estimated multi-trait BV:

$${\text{RFI}}_{\text{Index}:i}={\text{BV}}_{\text{DMI}:i}\;–\;\left(0.456\;\times {\text{BV}}_{\text{ECM}:i}+0.0508\times {\text{BV}}_{\text{MBW}:i}\;–\;3.25\times {\text{BV}}_{\text{BWL}:i}+3.25\times {\text{BV}}_{\text{BWG}:i}\right),$$ (3)

where BV_t:i_ is either the estimated PEBV or GEBV for the trait t and animal i and the index weight values are the same values as the energy requirement values applied for the ReFI_RDC_ metric.

#### RZFeedEfficiency

The recently published German FE index for Holstein dairy cows (Equation 4) differs from the RFI_Index_ metrics in that it does not account for the feed requirement for maintenance; therefore, it resembles a simplified version of the feed saved index proposed by Pryce et al. (2015). The German index is called RZFeedEfficiency (RZFE; Abdalla et al., 2024), and in this study, it was calculated for an animal i by the same procedure as described for the RFI_Index_ but by applying weights that are used in the German index, as follows:

$${\text{RZFE}}_{i}=\left(0.4\;\times {\text{BV}}_{\text{ECM}:i}\;–\;4.5\times {\text{BV}}_{\text{BWL}:i}+4.5\times {\text{BV}}_{\text{BWG}:i}\right)\;–\;{\text{BV}}_{\text{DMI}:i},$$ (4)

wherein, in addition to the differences with RFI_Index_, the index weights are also reversed, and thus, higher positive RZFE values are favorable and indicate higher efficiency.

#### Residual feed intake

RFI is the fifth metric (Equation 5), which is a common form of a one-step RFI model, and it was applied as follows:

$${\text{DMI}}_{\text{himwp}}=c1\times {\text{ECM}}_{\text{iw}}+c2\times {\text{MBW}}_{\text{iw}}+c3\times {\text{BWL}}_{\text{iw}}+c4\times {\text{BWG}}_{\text{iw}}+{\text{HPY}}_{h}+{\text{HTM}}_{m}+{\text{pe}}_{i}+u_{i}+e_{\text{himwp}},$$ (5)

where DMI_himwp_ is a DMI observation of cow i in lactation week w recorded in HPY h and HTM m; c1–c4 are the partial regression coefficients for the regression of DMI on the energy sinks. The model effects had the same number of effect levels as given in model (1). The HPY effect was modeled as a fixed effect, and the random HTM_m_, pe_i_, u_i_, and e_himwp_ effects were modeled in the same way as in model (2) but with a univariate analysis.

#### Energy conversion efficiency

It is an ECE trait used for benchmarking the five studied feed-utilization efficiency metrics, where ECE does not involve any partial regression coefficients and represents a clearly established FE trait. The model used for the analysis of ECE (Equation 6) included the same fixed and random effects as in model (5), with the exception that the energy sinks in model (5) were replaced with a fixed effect for lactation week:

$${\text{ECE}}_{\text{himwp}}={\text{LWK}}_{w}+{\text{HPY}}_{h}+{\text{HTM}}_{m}+{\text{pe}}_{i}+u_{i}+e_{\text{himwp}},$$ (6)

where ECE_himwp_ is the ECE observation of cow i in lactation week w recorded in HPY h and HTM m, LWK_w_ is the fixed effect of lactation week w, and all other effects in the model were specified in the same way as in model (5) but with a univariate analysis.

#### Estimation of variance components and the prediction of breeding values

The required variance components were estimated using pedigree BLUP models and a Monte Carlo expectation-maximization REML algorithm (Matilainen et al., 2012). The heritabilities and variances for the RFI_index_ and RZFE metrics were derived based on the variance components estimated for model (2) and the applied index weights of the metrics. The models developed were used to predict the necessary PEBV and GEBV for forward validation, as described in the following chapter (see the Model validation section). For the estimation of GEBV, we carried out single-step GBLUP analyses by substituting the inverse of the numerator relationship matrix **A** ^–1^ with the inverse of the single-step relationship matrix **H** ^−1^ in the BLUP models (Aguilar et al., 2010; Christensen and Lund, 2010), i.e., **H** ^−1^ = **A** ^–1^ + $\left[\begin{array}{cc} 0 & 0\\ 0 & G^{-1}-A_{22}^{-1} \end{array}\right]$ , where **G** ^–1^ is the inverse of the weighted genomic relationship matrix, **G** = G* × 0.9 + **A**_22_ × 0.1, and $A_{22}^{-1}$ is the inverse of the numerator relationship matrix for the genotyped animals. Method 1 in VanRaden (2008) was used to construct genomic relationship matrix G*. The Relax2 program (Strandén and Vuori, 2006) was used for pruning and validating the pedigree, the Htginv_v7 program (Strandén and Mäntysaari, 2018) was used to construct the **H** ^−1^ matrix, and MiX99 software suite (Pitkänen et al., 2022) was used for estimating BV.

#### Model validation

To assess the predictive ability of the different metrics, the linear regression forward validation method described by Legarra and Reverter (2018) (LR) was used. In this method, PEBV and GEBV from the full dataset (PEBV_f_ and GEBV_f_) are regressed on PEBV and GEBV from the reduced dataset (PEBV_r_ and GEBV_r_), respectively, as follows: (P)GEBV_f_ = b0 + b1 × (P)GEBV_r_, where b0 and b1 are the bias (intercept) and dispersion (regression coefficient), respectively, and the correlation between (P)GEBV_f_ and (P)GEBV_r_ is the prediction accuracy (PAC). The 88 youngest genotyped animals, born between 2017 and 2019, which had records in the full dataset but neither records nor daughters with records in the reduced dataset, were included in the validation group.

The Delta method was applied to calculate the standard errors of heritabilities (h^2^) and repeatabilities (r). In addition, the ordinary nonparametric bootstrap method (R Development Core Team, 2012) was applied to calculate the standard errors of estimates for different metrics to assess the consistency of the estimates. Furthermore, Pearson and Spearman correlations between GEBV of animals with phenotypes for the different metrics were calculated. The ECE phenotypes were adjusted for all the effects estimated with model (6) except for the animal additive genetic effect to obtain adjusted phenotypes that are equivalent to ECE yield deviation, and the correlations between these adjusted ECE phenotypes and the cows’ GEBV for the different metrics were calculated.

## Results and discussion

### Summary statistics and partial regression coefficients

Descriptive statistics for the phenotypes of the studied feed efficiency data and the applied alternative eDMI values are provided in Table 1. On average, a cow consumed 19.4 kg DM/d containing 212.7 MJ ME and produced 29.0 kg ECM with 4.43% milk fat and 3.63% milk protein. The average energy density of the diets was 10.94 MJ ME per kg DM, which is consistent with reports from previous studies (Mäntysaari et al., 2012; Karlsson et al., 2022). The averages of eDMI using different energy requirement formulations were relatively similar. When applying the RFI model, the average of eDMI was the closest to the average of the observed DMI. In contrast, the formulation recommended in the National Academies of Sciences, Engineering, and Medicine (2021) overestimated the DMI by 1.4 kg. The differences are due to differences in the regression coefficients of energy sinks between different formulations. For instance, a lower requirement coefficient has been allocated to ECM (0.229 vs. 0.276 for RFI) and a higher requirement coefficient to MBW (0.108 vs. 0.068 for RFI) in the National Academies of Sciences, Engineering, and Medicine (2021) compared to that of the other formulations. In addition, the National Academies of Sciences, Engineering, and Medicine (2021) has a requirement coefficient for BCS in the formula (−0.689), which makes it somewhat different from the other formulations. Moreover, the prediction formula for DMI presented in the National Academies of Sciences, Engineering, and Medicine (2021) was developed for Holstein cows and used observations from both primiparous and multiparous cows, which may have different metabolism and energy requirements. The SD of the eDMI was the largest for the formulation that was developed for the RDC FE data, which is most likely because, among all studied formulations, it had the largest energy requirement coefficient for ECM.

The partial regression coefficients for the different metrics are summarized in Table 2. To make the partial regression coefficients of the different metrics comparable, we converted the coefficients of the eDMI prediction equation from the National Academies of Sciences, Engineering, and Medicine (2021) to the ECM and MBW scales. Assuming that 1 kg of ECM has 0.75 Mcal NEL, it leads to a ReFI_NRC2021_ coefficient of 0.229 kg DM per kg ECM. The average MBW in our data was 119.7 kg^0.75^, which corresponds to a BW of 590 kg. Using the coefficient of 0.022 kg DM/kg BW from equation 2-1 in the National Academies of Sciences, Engineering, and Medicine (2021) resulted in 0.108 as the coefficient for MBW. However, apart from the National Academies of Sciences, Engineering, and Medicine (2021) formulation applied in this study, the National Academies of Sciences, Engineering, and Medicine (2021) proposed increasing the energy requirement for maintenance by 25% compared to NRC (2001), which would result in a coefficient of 0.121 for MBW.

**TABLE 2:** Partial regression coefficients used in different metrics.

Metric	ECM	MBW	BWL	BWG	BCS
ReFI_RDC_	0.456	0.051	−3.25	3.25	—
ReFI_NRC2021_ ^a^	0.229^b^	0.108^c^	—	—	−0.689
RFI_Index_	0.456	0.051	−3.25	3.25	—
RZFE	0.400	—	−4.50	4.50	—
RFI	0.276	0.068	−3.80	1.44	—

ECM, energy-corrected milk; MBW, metabolic body weight (BW^0.75^); BWL, body weight loss; BWG, body weight gain; BCS, body condition score; ReFI_RDC_ = regression on expected feed intake using expected DMI from tailored coefficients; ReFI_NRC2021_ = regression on expected feed intake using expected DMI from NASEM (2021); RFI_Index_ = multi-trait residual feed intake; RZFE = German feed efficiency index; RFI = single step residual feed intak.

^a^ Expected DMI for this scenario was calculated using the DMI predicting equation 2-1 from the National Academies of Sciences, Engineering, and Medicine (2021) with coefficients that correspond to the values given here.

^b^ This was calculated by assuming that 1 kg of ECM has 0.752 Mcal NEL according to equation 2-1 in the National Academies of Sciences, Engineering, and Medicine (2021).

^c^ Average MBW in our data was 119.7, which corresponds to a BW of 590 kg. Using the coefficient from equation 2-1 resulted in 0.108 as the coefficient for MBW.

When comparing the partial regression coefficients of the formulations that were developed by considering nutritional studies (ReFI_RDC_, RFI_Index_, and RZFE) with those that are based on partial regression analyses (ReFI_NRC2021_ and RFI), it can be seen that formulations based on partial regression analyses provide lower regression coefficients for ECM and higher regression coefficients for MBW. The partial regression coefficients obtained by applying the one-step RFI model were 0.276 for ECM and 0.068 for MBW, while the corresponding values based on RDC data were 0.456 and 0.0508, respectively. The requirement formulations for ReFI_RDC_ and RZFE differ substantially in the allocation of feed toward milk production and growth. For ReFI_RDC_, the coefficient for BWG is 7.1 times larger than that for ECM. In contrast, for RZFE, the coefficient for BWG is 11.3 times larger than that for ECM, implying that the lower conversion efficiency of DMI to body tissue has been considered in the German RZFE metric.

### Variance components

Variance components, h^2^, and repeatability for the five feed-utilization efficiency metrics and for ECE are provided in Table 3. Combined residual variance estimates, calculated as the weighted averages of the residual estimates for the five lactation periods, were used to facilitate comparisons. Among the five metrics, the lowest residual variance was observed for RFI_Index_, followed by similar estimates for RZFE, ReFI_NRC2021_, and RFI, with the highest estimate for ReFI_RDC_. Nevertheless, heritabilities were on the same level for RFI, ReFI_RDC_, and RFI_Index_, (0.23, 0.24, and 0.26, respectively) and slightly higher for RZFE and ReFI_NRC2021_ (0.29 and 0.30, respectively). This may be due to the estimated genetic variance for ReFI_RDC_ (0.00303 × 20^2^ = 1.21 kg^2^), which was higher than those estimated for RFI and RFI_Index_. We obtained the highest additive genetic variance estimate for ReFI_NRC2021_ (0.00334 × 20.8^2^ = 1.45 kg^2^), followed by that for RZEF (1.41 kg^2^). The estimated repeatabilities were rather similar for ReFI_RDC_, ReFI_NRC2021_, RFI_Index_, and RZFE (0.50–0.52) but slightly lower for RFI (0.48). Due to the size of the available data, the heritability and reliability estimates were associated with large standard errors.

**TABLE 3:** Estimates of the genetic, residual, permanent environmental (Var_G_, Var_R_, and Var_PE_, respectively), variances, heritability (h^2^), and repeatability (r) for regression of feed intake calculated with different criteria using a repeatability animal model.

Metric	Var_PE_	Var_G_	Var_R_*	h^2^_S.E._	r _S.E._
ReFI_RDC_	0.00333	0.00303	2.502	0.24 _0.20_	0.50 _0.10_
ReFI_NRC2021_	0.00244	0.00334	2.348	0.30 _0.21_	0.52 _0.11_
RFI_Index_	1.109	1.137	2.166	0.26 _NA_	0.51 _NA_
RZFE	1.078	1.409	2.330	0.29 _NA_	0.52 _NA_
RFI	1.127	1.043	2.369	0.23 _0.20_	0.48 _0.11_
ECE	0.000118	0.000081	0.000157	0.23 _0.21_	0.56 _0.09_

Var_PE_, permanent environmental variance; Var_G_, additive genetic variance; Var_R_, residual variance; h^2^, heritability; r, repeatability; ReFI_RDC_ = regression on expected feed intake using expected DMI from tailored coefficients; ReFI_NRC2021_ = regression on expected feed intake using expected DMI from NASEM (2021); RFI_Index_ = multi-trait residual feed intake; RZFE = German feed efficiency index; RFI = single step residual feed intake; ECE = energy conversion efficiency.

*There were five lactation periods, and the weighted average of the residual variances for each approach were calculated and subsequently used to calculate heritabilities; NA, not available.

Reports on h^2^ of RFI in dairy cows from different countries agreed well with the estimates from the present study, which ranged from 0.16 to 0.38 (Pryce et al., 2012; Lu et al., 2015; Stephansen et al., 2022). Similar values of h^2^ have been reported for Holstein dairy heifers (0.24) (Khanal et al., 2023), Nellore cattle (0.21) (Brunes et al., 2023), and other species such as turkeys (0.15) (Abdalla et al., 2019) and pigs (0.30) (Wang et al., 2022). The magnitude of the estimated genetic variance and h^2^ indicates that sufficient genetic variation for FE exists, and these traits can be improved through selection.

The reason for the higher h^2^ estimate for ReFI_NRC2021_ compared to that for ReFI_RDC_ is unclear. The energy requirement formulation applied for ReFI_NRC2021_ allocates less DMI to ECM and more DMI toward BW compared to the energy requirement formulation for ReFI_RDC_. If the difference between the observed and expected DMI is less influenced by the actual difference in FE and more affected by inappropriate coefficients, it may inflate heritability as the difference may capture variations caused by sink traits rather than by variations in FE. The higher h^2^ estimate for RZFE may be due to the fact that RZFE is an index trait composed of a metabolic efficiency component, similar to the other metrics in this study, and of a maintenance component that accounts for the size of the cow.

The ECE trait, which was used for validation purposes in this study, had a relatively similar h^2^ to other FE metrics. Due to the limited availability of both DMI records and assays of ME content, estimates of ECE are scarce in the literature. Performing a longitudinal analysis, Hurley et al. (2017) estimated h^2^ for ECE to be between 0.06 in DIM 60 to 0.28 in DIM 250. Liinamo et al. (2015) reported lactational h^2^ of ECE to be 0.16 for RDC cows. The estimated h^2^ value for gross feed efficiency was 0.32 for Holstein cows at Iowa State University’s Research Farm (Spurlock et al., 2012). In another study using a larger dataset, Vallimont et al. (2011) reported an h^2^ value of 0.14 for DMI efficiency, which was the ratio of fat-corrected milk to DMI.

### Validation of metrics

Forward validation results obtained using the LR procedure are presented in Table 4. The results show that the biases obtained from pedigree- and genomic-based prediction models differed, indicating lower biases for ReFI_RDC_, ReFI_NRC2021_, and ECE and higher biases for the index metrics RFI_Index_ and RZFE. Among the different metrics, RFI_Index_, RZFE, and RFI showed higher prediction bias for both pedigree- and genomic-based predictions, ranging between 0.09 and 0.22 genetic standard deviations. The dispersion estimates for the pedigree- and genomic-based prediction models were lower than 1.0. Among the genomic models, the lowest dispersion estimate (0.454) was obtained for RFI_Index_ and the highest (0.951) was for RZFE. In addition, dispersions estimated using genomic predictions were generally associated with lower standard errors, indicating greater stability of the estimates. Correlations between GEBV_f_ and GEBV_r_ for different metrics ranged from 0.30 to 0.53 and were significantly higher than the correlations between PEBV_f_ and PEBV_r_ (from 0.17 to 0.41; Table 4). Among the metrics, ReFI_RDC_ yielded the highest PAC, followed by RFI. Although ReFI_RDC_ and ReFI_NRC2021_ had the same modeling, ReFI_RDC_ yielded 23% higher PAC. This can be attributed to the applied regression coefficients in the calculation of eDMI, which differed considerably (Table 2). The applied regression coefficients for eDMI in ReFI_RDC_ are derived from nutritional studies and reflect the DMI requirement for ECM, MBW, and BW change. On the contrary, the regression coefficients applied in calculating eDMI for ReFI_NRC2021_ were obtained from the regression analyses reported by the National Academies of Sciences, Engineering, and Medicine (2021). Partial regression analyses ensure good model fit in modeling the total energy requirement. Nevertheless, a partial coefficient for a specific energy sink might be suboptimal, which reduces the accuracy of the individual eDMI values. For instance, for ReFI_NRC2021_, the applied coefficient for ECM underestimates the expected feed requirement for ECM. Furthermore, the coefficient of variation of ECM is higher than that of MBW, which enables ReFI_RDC_ to capture a greater proportion of total variation in the composite trait and, thus, better stabilize the training and validation GEBV and ensure that they are on the same scale. On the other hand, ReFI_RDC_ includes BWL and BWG, which are measured more accurately compared to the subjective nature of BCS in the National Academies of Sciences, Engineering, and Medicine (2021). In addition, for ECE, the correlation between GEBV_f_ and GEBV_r_ was considerably higher than that between PEBV_f_ and PEBV_r_ (0.43 vs. 0.18). Generally, FE metrics with single-step modeling led to higher PAC.

**TABLE 4:** Results of forward validation by the Legarra–Reverter procedure (regression of PEBV and GEBV predicted using the full dataset on PEBV and GEBV predicted using the reduced dataset, respectively).

Metric^a^	Forward validation parameter			
	Model^b^	b0	b1	corr
ReFI_RDC_	BLUP	0.007 _0.002_	0.724 _0.137_	0.41 _0.065_
	ssGBLUP	−0.001 _0.002_	0.638 _0.109_	0.53 _0.069_
ReFI_NRC2021_	BLUP	−0.003 _0.003_	0.514 _0.165_	0.30 _0.053_
	ssGBLUP	0.003 _0.002_	0.518 _0.117_	0.43 _0.072_
RFI_Index_	BLUP	−0.171 _0.001_	0.479 _0.001_	0.17 _0.030_
	ssGBLUP	−0.244 _0.001_	0.454 _0.002_	0.33 _0.055_
RZFE	BLUP	0.234 _0.001_	0.951 _0.001_	0.30 _0.062_
	ssGBLUP	0.232 _0.001_	0.735 _0.004_	0.40 _0.060_
RFI	BLUP	−0.149 _0.037_	0.547 _0.168_	0.30 _0.052_
	ssGBLUP	−0.097 _0.035_	0.605 _0.116_	0.47 _0.072_
ECE	BLUP	−0.0004 _0.0004_	0.552 _0.333_	0.18 _0.030_
	ssGBLUP	−0.0007 _0.0004_	0.747 _0.161_	0.43 _0.061_

^a^ ReFI_RDC_ = regression on expected feed intake using expected DMI from tailored coefficients; ReFI_NRC2021_ = regression on expected feed intake using expected DMI from NASEM (2021); RFI_Index_ = multi-trait residual feed intake; RZFE = German feed efficiency index; RFI = single step residual feed intake; ECE = energy conversion efficiency.

^b^ BLUP and ssGBLUP are regressions of PEBV and GEBV using the full dataset on PEBV and GEBV using the reduced dataset, respectively.

The results indicate that including genomic information in the evaluation of animals leads to higher PAC. Using different approaches (GBLUP and BayesA), Pryce et al. (2012) reported the average PAC for RFI values of 0.37 and 0.31 in Australian and New Zealand dairy heifers, respectively. In addition, Li et al. (2020) estimated an average PAC of 0.34 for RFI for all phenotyped animals using single-step GBLUP in their study. In another study, Abdalla et al. (2019) reported improved dispersion and higher PAC using single-step GBLUP compared to PBLUP for RFI in turkey. The highest attainable PAC using genomic selection may vary depending on the specific application and data used. In general, it can range from approximately 0.4 to 0.9, implying that between 40% and 90% of the genetic variance in a trait can be explained by genomic data.

Despite the LR forward prediction method allowing the assessment of the quality of predicted BVs for the metrics compared in this study, it does not allow the assessment of the metrics’ potential for selecting the overall feed-efficient animals. Therefore, we assess the correlations between the GEBV of the different metrics with genomic breeding values for ECE. In summary, ECE is easy to calculate and understand and is the actual FE trait that dairy farmers want to improve. Pearson and Spearman correlations between GEBV of animals for different residual metrics and ECE are shown in Table 5. The GEBV for ReFI_RDC_ and RFI_Index_ had the highest Pearson correlations with the GEBV for ECE (−0.70 and −0.69, respectively) and the highest Spearman correlations, whereas the GEBV for RFI_NRC2021_ and RFI had the lowest Pearson correlation with GEBV for ECE (−0.54 and −0.58, respectively). This implies that the selection of individuals with lower GEBV for ReFI_RDC_ or RFI_Index_ would best improve the overall FE compared to selection based on GEBV, which is based on the other studied metrics. Contrary to our expectation, the correlation between ReFI_RDC_ and RFI_Index_ was smaller than that between ReFI_RDC_ and RFI (0.82 vs. 0.93), and it deviated from unity, although both used the same set of partial regression coefficients. This is because of the modeling, as ReFI_RDC_ and RFI were performed in a single-step manner, while for RFI_Index_, we first predicted multi-trait (G)EBVs and then combined them.

**TABLE 5:** Pearson (above diagonal) and Spearman (below diagonal) correlations (S.E. are subscripted) between GEBV of the animals with phenotype (n = 791) for different metrics of feed-utilization efficiency.

Metric^a^	ReFI_RDC_	ReFI_NRC2021_	RFI_Index_	RZFE	RFI	ECE
ReFI_RDC_	​	0.84 _0.02_	0.82 _0.02_	−0.75 _0.03_	0.93 _0.01_	−0.70 _0.02_
ReFI_NRC2021_	0.80 _0.02_	​	0.84 _0.02_	−0.69 _0.03_	0.92 _0.02_	−0.54 _0.03_
RFI_Index_	0.77 _0.03_	0.78 _0.03_	​	−0.92 _0.02_	0.88 _0.02_	−0.69 _0.03_
RZFE	0.67 _0.03_	0.60 _0.03_	0.88 _0.02_	​	−0.84 _0.02_	0.62 _0.03_
RFI	0.91 _0.01_	0.90 _0.02_	0.85 _0.02_	0.78 _0.03_	​	−0.58 _0.03_
ECE	0.66 _0.03_	0.50 _0.03_	0.65 _0.03_	0.55 _0.03_	0.54 _0.03_	​

^a^ ReFIRDC = regression on expected feed intake using expected DMI from tailored coefficients; ReFINRC2021 = regression on expected feed intake using expected DMI from NASEM (2021); RFIIndex = multi-trait residual feed intake; RZFE = German feed efficiency index; RFI = single step residual feed intake; ECE = energy conversion efficiency.

The latter findings are also supported by the correlations between ECE phenotypes that are adjusted for all effects in the model except for the animal additive genetic effect and the GEBV of different metrics (Table 6). Among the studied metrics, the animals’ GEBV for ReFI_RDC_ had the highest correlation (−0.64), and GEBV for RFI_NRC2021_ (−0.47) had the lowest correlation with the adjusted phenotypes of ECE. The reason for the correlation of ECE being higher with ReFI_RDC_ compared to that with RZFE requires further analysis. It could be expected that RZFE has a higher correlation with ECE because the RZFE index includes the DMI needed for maintenance as an additional component, and thus, it should better resemble ECE. One reason could be that the applied energy requirement coefficient for ECM in the RZFE metric is rather low (0.4) and corresponds to a very high efficiency of using ME for lactation of *k*_*l*_ = 0.72, which most likely has not been achieved by the cows. On the other hand, ReFI_RDC_ is based on a random regression approach and, therefore, has the same capability as ECE to account for the multiplicative effect of efficiency factors (Lidauer et al., 2023). GEBV for RFI_NRC2021_, showing a relatively low correlation with the adjusted ECE observations (−0.47), is most likely due to the very low energy requirement coefficient for ECM, which might be in poor agreement with the DMI requirement for production. Therefore, considering an animal’s specific energy requirements for various energy sinks, along with the choice of an appropriate model, is essential when aiming to maximize the genetic gains for FE in a breeding program. Although all the observations in this study were from primiparous cows, as the calculations were based on the unit of energy sinks, it can be expected that similar results would also be obtained for multiparous cows.

**TABLE 6:** Correlations (S.E. are subscripted) between ECE phenotypes adjusted for all effects in the model except the animal additive genetic effect and GEBV of different metrics (n = 791).

Metric^a^	Correlation
ReFI_RDC_	−0.64 _0.03_
ReFI_NRC2021_	−0.47 _0.03_
RFI_Index_	−0.58 _0.03_
RZFE	0.52 _0.03_
RFI	−0.49 _0.03_
ECE	0.88 _0.02_

^a^ ReFIRDC = regression on expected feed intake using expected DMI from tailored coefficients; ReFINRC2021 = regression on expected feed intake using expected DMI from NASEM (2021); RFIIndex = multi-trait residual feed intake; RZFE = German feed efficiency index; RFI = single step residual feed intake; ECE = energy conversion efficiency.

## Conclusion

Different FE metrics based on various models and energy requirement systems were compared in this study. Genomic information was also included to assess the extent of improvement in prediction accuracy that could potentially be achieved by implementing single-step GBLUP. The results indicated that both differences in energy requirement systems and differences in the modeling process would lead to different rankings of animals varying in their feed-utilization efficiency. When using the same model, ReFI_RDC_ outperformed ReFI_NRC2021_, yielding 23% higher prediction accuracy, which underscores the importance of choosing proper partial regression coefficients (or predicting equations) to calculate the expected DMI. On the other hand, when using the same partial regression coefficients but different modeling processes, ReFI_RDC_ led to higher prediction accuracy and showed a slightly higher correlation with ECE compared with RFI_Index_. The results indicate that modification in the modeling of FE traits would lead to a significant genetic gain. It also indicates that considerable improvement in FE could be achieved even when a small number of phenotyped and genotyped animals are available. However, a larger reference population is needed to achieve higher prediction reliabilities and accelerate the genetic improvement for FE.

## Data Availability

The dataset used in this study (obtained from two research farms in Jokioinen, one in Helsinki and one in Maaninka) belong to Natural Resources Institute (LUKE) and hence, are not publicly available. Data are however available upon required agreement with LUKE and should be directed to the last author Martin H. Lidauer, martin.lidauer@luke.fi.
